# Knowledge and Awareness About Gastric Cancer Among the General Population in Al-Baha City, Saudi Arabia

**DOI:** 10.7759/cureus.39589

**Published:** 2023-05-28

**Authors:** Ali G Alghamdi, Alshareef M Alshareef, Aghnar T Alzahrani, Ziyad S Alharthi, Sarah S Alghamdi, Ahmed M Alghamdi, Faisal A Alzahrani, Reem A Alzahrani

**Affiliations:** 1 College of Medicine, Al-Baha university, Al-Baha, SAU

**Keywords:** risk factors for gastric cancer, h.pylori, albaha, knowledge, awareness, gastric cancer

## Abstract

Background

Gastric cancer is a significant health concern worldwide, and its incidence varies across different populations. This study aimed to assess the level of knowledge and awareness of gastric cancer among the general population in Al-Baha City, Saudi Arabia.

Methodology

This is a cross-sectional study that was conducted among the residents of Al-Baha city older than 18 years. The study was conducted based on a questionnaire that has been developed by a previous study. Data were initially recorded in an Excel sheet before being exported to the SPSS program, version 25 for data analysis.

Results

The survey included 426 respondents from Al-Baha city, Saudi Arabia, with 56.8% being females and the majority being in the age groups (21-30 years). Alcohol consumption (mean=4.5, SD= 0.77), smoking cigarettes or Shisha (mean= 4.38, SD=0.852), family history of gastric cancer (mean= 4, SD=1.008), a past medical history of gastric cancer (mean= 3.99, SD=0.911), stomach ulcer (mean=3.76, SD=0.898), and consumption of smoked food (mean= 3.69, SD=0.956) are the most widely recognized risk factors. The most highly recognized symptoms are gastrointestinal bleeding (mean= 4.03, SD=0.875), abdominal lump (mean= 3.94, SD=0.926), weight loss (mean= 3.93, SD=0.963), recurrent nausea and vomiting (mean=3.76, SD=0.956), and abdominal pain (mean= 3.57, SD=0.995). The study also identified several subgroups of the population that may benefit from targeted educational programs, including individuals in the age group of 41-50 years and those in non-medical occupations.

Conclusion

The study found that participants had a moderate level of knowledge about the risk factors and symptoms of gastric cancer, with significant variability among different subgroups of the population. Further research is needed to investigate the prevalence and risk factors of gastric cancer in Saudi Arabia and other similar populations, to develop effective prevention and management strategies for this disease.

## Introduction

Gastric cancer is the fourth most common malignancy worldwide with an estimated incidence of 989,600 new cases in 2008 and remains the second cause of death of all malignancies worldwide [1]. It is known to be an aggressive disease with poor treatment outcomes [2]. In Saudi Arabia, the incidence of gastric cancer was reported to be 2.7% in 2013, and it was ranked the 12th and the 9th most common cancer in Saudi women and men respectively [3]. 

The early stages of gastric cancer may not present any symptoms, making early detection and treatment challenging. However, as the disease advances, symptoms such as abdominal pain, nausea, vomiting, and weight loss may occur. Early detection and treatment are critical to improving outcomes for patients with gastric cancer. Early gastric cancer has a significantly better prognosis; however, the 4-year survival rate of gastric cancer is significantly low [4]. This could be due to poor public knowledge and awareness of the risk factors contributing to gastric cancer and the warning signs of gastric cancer [5]. Risk factors for gastric cancer include smoking, alcohol intake, increased salt intake, and pickled food as well as atrophic gastritis and *Helicobacter pylori* infection [6]. Prevention and management strategies for gastric cancer include identifying and managing risk factors, such as *H. pylori* infection and chronic inflammation of the stomach lining, adopting healthy lifestyle choices, such as quitting smoking and reducing alcohol consumption, and regular screening and diagnostic tests.

Assessing the level of awareness of the general public about gastric cancer is crucial in developing effective prevention and management strategies for the disease. Several studies have been conducted to assess the awareness of gastric cancer among the general public in different regions and populations [7-9]. One study conducted in Saudi Arabia found that the level of awareness of gastric cancer was relatively low among the general public, with disease awareness only among 31% of the participants. The study also found that the level of awareness was significantly lower among women, younger age groups, and individuals with lower educational levels [7]. Another study conducted in Iran found that the level of awareness of gastric cancer among the general public was low, with only 16.8% of the participants being aware of the disease. The study also found that there was a significant lack of knowledge about the risk factors and symptoms of gastric cancer, highlighting the need for targeted educational interventions to increase awareness and understanding of the disease [8]. Similarly, a study conducted in China found that the level of awareness of gastric cancer was low among the general public, with only 35.5% of the participants having ever heard of the disease. The study also found that there was a significant lack of knowledge about the risk factors and symptoms of gastric cancer, emphasizing the need for targeted educational interventions to improve awareness and understanding of the disease [9]. The primary aim of this study is to assess the knowledge of the risk factors and warning symptoms of gastric cancer among Al-Baha Residents.

## Materials and methods

This is a cross-sectional study that was conducted using a pretested questionnaire among all the residents of Al-Baha city older than 18 years. The sample is selected randomly [10] in which experienced interviewers (data collectors) based in Al-Baha city ask the individual citizens of Al-Baha city face-to-face to take part in our study and explain the importance of the questionnaire to them. Selection bias [11] was avoided through the use of interviewers to gather the information from the participants face-to-face instead of the online-based questionnaire, so we don’t miss groups with limited access to the internet such as elderly and lower-income households. The interviewers were designated to take responses from different areas in Al-Baha city, including but not limited to hospitals, malls, colleges, and campaigns so that each individual in the population had an equal chance of being selected. 

The inclusion criteria included residents of Al-Baha city who were 18 years or older, while non-Al-Baha residents, those who were younger than 18 years old, and people who refuse to take part in the study\incomplete questionnaires were excluded from the study. 

A minimum sample size [12] was calculated using the standard sample size formula at a confidence interval of 95% and a minimum sample of 384 was found appropriate for our study; the final sample size acquired was 426. The data collection was conducted for a duration of two months. 

Many of the items included in the questionnaire used for the present study were sourced from a similar study in China [13]. The original version of the questionnaire was in English but was translated into Arabic through an expert translator for data collection. The Arabic version was then back-translated to the English version by another independent translator who was blinded to the original English version. 

The questionnaire included four sections; the first section is related to the sociodemographic data (gender, age, nationality, residence, marital status, educational level, occupation, monthly income, personal history of GI diseases, past medical history or family history of gastric cancer), the second part had items that assessed the knowledge of risk factors related to gastric cancer, the third part had items that assessed the knowledge of the warning symptoms of gastric cancer, and the fourth part included items used to assess the knowledge of the participants towards the cure and prevention of gastric cancer (see Appendix).

Before the start of data collection, a pilot study was done on 100 adults to test the applicability and comprehension of the survey. No modifications were needed as the questions were sufficiently understandable. The study was approved by the Scientific Research & Ethics committee, Al-Baha University, Faculty of Medicine, Approval number: REC\SUR\BU-FM\2023\11, and the data were collected between January and March 2023.

Data entry and analysis

Data were initially recorded in an Excel sheet before being exported to the SPSSS program, version 25. Then, the Cronbach alpha coefficient was used to evaluate the reliability of the tool. For the categorical variables, descriptive analysis in the form of frequencies and percentages was performed. The mean and standard deviation were used to describe data for quantitative variables. The risk factor knowledge score was obtained by adding all the points from the risk factor statements multiplying by 80 and dividing by 100. The knowledge score for symptoms was obtained by adding all of the points from the symptom statements multiplying by 30 dividing by 100. The t-test and ANOVA were used in a univariate analysis to investigate variations in mean knowledge. To predict factors influencing participants' knowledge, multivariate linear regression analysis was employed. The confidence interval was set at 95%, and a margin of error of 0.05 is considered statistically significant.

Cronbach's alpha coefficient reliability test for the study domains was illustrated in the table below (Table 1). The values of 0.875, 0.844, and 0.908 were obtained for the knowledge about risk factors, knowledge about symptoms, and the overall statements respectively. These numbers indicate that the tool is reliable, as all the numbers are above 0.70 as previously reported.

**Table 1:** Test of Reliability

Domain	No. of statements	Cronbach alpha
Knowledge about risk factors of gastric cancer	16	0.875
Knowledge about symptoms of gastric cancer	6	0.844
Overall	22	0.908

## Results

The survey included 426 respondents from Al-Baha city, Saudi Arabia, with 56.8% being females and the majority being in the age groups (less than or equal to 20 years) and (21-30 years) with 17.6% and 39.9%, respectively. Almost all are Saudi (99.5%), and the majority have a college education or more (69.0%), with 41.8% being students. More than a third of the participants (33.8%) are students or work in the medical area, and more than fifty (57.3%) earn less than 5,000 per month. The outcomes are shown in Table (2). 

**Table 2:** Baseline characteristics of the study participants

Variable	N	%
Gender:		
male	184	43.2
female	242	56.8
Age Mean± SD (Min- Max) 32.2 ± 13.3 (15- 80):		
<=20years	75	17.6
21-30 years	170	39.9
31-40years	65	15.3
41-50years	69	16.2
>50years	47	11.0
Nationality:		
Non-Saudi	2	0.5
Saudi	424	99.5
Education:		
Illiteracy	4	0.9
elementary school	8	1.9
intermediate school	8	1.9
high school	112	26.3
college or above	294	69.0
Occupation:		
student	178	41.8
employed	111	26.1
unemployed	91	21.4
Retired	36	8.5
freelancer	10	2.3
Do you work \ Study in a medical Field?		
no	282	66.2
yes	144	33.8
Monthly income:		
less than 5000	244	57.3
5000-10000	80	18.8
10000-20000	88	20.7
more than 20000	14	3.3

Table 3 shows a descriptive analysis of family and medical histories. A total of 15.5% of the participants have a history of upper or lower GI tract disease (peptic ulcer, chronic gastritis, and inflammatory bowel disease). GERD, chronic gastritis, *H. pylori*, and stomach ulcers account for 18.2%, 18.2%, 31.8%, and 15.2% of all cases, respectively. Almost all (99.3%) had no prior medical history of stomach cancer, and only 4.9% have a family history of the disease.

**Table 3:** Family and medical history of gastric cancer GERD: Gastroesophageal reflux disease. H.pylori: Helicobacter pylori IBS: irritable bowel syndrome SMA syndrome: superior mesenteric artery syndrome

Variable	N	%
Do you have any disease of the upper or lower GI tract “peptic ulcer, chronic gastritis, inflammatory bowel diseases etc....		
no	360	84.5
yes	66	15.5
If the answer to the previous question is yes, what’s the diagnosis? N= 66		
GERD	12	18.2
chronic gastritis	12	18.2
H. pylori	21	31.8
Gastric ulcer	10	15.2
IBS	4	6.1
SMA syndrome	1	1.5
Dyspepsia	4	6.1
Cholecystitis	1	1.5
Lymphoma	1	1.5
Past Medical History of Gastric Cancer		
no	423	99.3
yes	3	0.7
Family History of Gastric Cancer		
no	405	95.1
yes	21	4.9

Alcohol consumption (mean=4.5), smoking cigarettes or Shisha (mean= 4.38), family history of gastric cancer (mean= 4), a past medical history of gastric cancer (mean= 3.99), stomach ulcer (mean=3.76), and consumption of smoked food (mean= 3.69) are the most widely recognized risk factors. Table 4 shows that the less recognized risk factors which include a salty diet (mean= 3.34), pickled food consumption (mean= 3.32), male sex (mean= 3.26), frequent eating of leftovers (mean= 3.10), and frequent midnight snacking (mean= 2.88).

**Table 4:** Participants' Knowledge about risk factors of gastric cancer SD: Strongly disagree, D; disagree, N: Neutral, A: agree, SA: Strongly agree

Statement	SD	D	N	A	SA	Mean	SD
Alcohol consumption is one of the risk factors	3 (0.7%)	4 (0.9%)	43 (10.1%)	105 (24.6%)	271 (63.6%)	4.50	0.771
Smoking “cigarettes or Shisha” is one of the risk factors	2 (0.5%)	16 (3.8%)	44 (10.3%)	119 (27.9%)	245 (57.5%)	4.38	0.852
Family history of gastric cancer is one of the risk factors	7 (1.6%)	36 (8.5%)	65 (15.3%)	158 (37.1%)	160 (37.6%)	4.00	1.008
Past medical history of gastric cancer is one of the risk factors	4 (0.9%)	26 (6.1%)	76 (17.8%)	183 (43%)	137 (32.2%)	3.99	0.911
Stomach ulcer is one of the risk factors	3 (0.7%)	37 (8.7%)	107 (25.1%)	193 (45.3%)	86 (20.2%)	3.76	0.898
Consumption of smoked food is one of the risk factors	8 (1.9%)	32 (7.5%)	124 (29.1%)	166 (39%)	96 (22.5%)	3.73	0.956
Atrophic gastritis is one of the risk factors	3 (0.7%)	31 (7.3%)	140 (32.9%)	175 (41.1%)	77 (18.1%)	3.69	0.876
Irregular\unhealthy diet is one of the risk factors	13 (3.1%)	44 (10.3%)	107 (25.1%)	171 (40.1%)	91 (21.4%)	3.66	1.021
Older age is one of the risk factors	6 (1.4%)	60 (14.1%)	109 (25.6%)	157 (36.9%)	94 (22.1%)	3.64	1.020
H. Pylori Infection is one of the risk factors	3 (0.7%)	56 (13.1%)	119 (27.9%)	164 (38.5%)	84 (19.7%)	3.63	0.966
Stress and anxiety is one of the risk factors	22 (5.2%)	53 (12.4%)	135 (31.7%)	123 (28.9%)	93 (21.8%)	3.50	1.117
A salty diet is one of the risk factors	17 (4%)	67 (15.7%)	157 (36.9%)	126 (29.6%)	59 (13.8%)	3.34	1.028
Consumption of pickled food is one of the risk factors	16 (3.8%)	55 (12.9%)	183 (43%)	121 (28.4%)	51 (12%)	3.32	0.971
Male sex is one of the risk factors	12 (2.8%)	57 (13.4%)	212 (49.8%)	97 (22.8%)	48 (11.3%)	3.26	0.926
Frequent eating of leftovers is one of the risk factors	30 (7%)	87 (20.4%)	161 (37.8%)	105 (24.6%)	43 (10.1%)	3.10	1.062
Frequent midnight snacking is one of the risk factors	42 (9.9%)	122 (28.6%)	150 (35.2%)	71 (16.7%)	41 (9.6%)	2.88	1.104

The most highly recognized symptoms\signs are gastrointestinal bleeding (mean= 4.03), abdominal lump (mean= 3.94), weight loss (mean= 3.93), recurrent nausea and vomiting (mean=3.76), and abdominal pain (mean= 3.57). The less recognized symptom is abdominal fullness (mean= 3.38) (Table 5). The participants have a mean knowledge of gastric cancer risk factors of 72.9, with a minimum score of 22.5 and a maximum score of 100. In addition, the participants have a mean knowledge of gastric cancer symptoms of 75.4 with a minimum score of 36.7 and a maximum score of 100 (Table 6).

**Table 5:** Participants’ knowledge about symptoms of gastric cancer SD: Strongly disagree, D; disagree, N: Neutral, A: agree, SA: Strongly agree

Statements	SD	D	N	A	SA	Mean	Std. Deviation
Gastrointestinal Bleeding is one of the warning symptoms	1 (0.2%)	21 (4.9%)	88 (20.7%)	171 (40.1%)	145 (34%)	4.03	0.875
Abdominal lump is one of the warning symptoms	2 (0.5%)	29 (6.8%)	97 (22.8%)	163 (38.3%)	135 (31.7%)	3.94	0.926
Weight Loss is one of the warning symptoms	3 (0.7%)	34 (8%)	92 (21.6%)	156 (36.6%)	141 (33.1%)	3.93	0.963
Recurrent nausea and vomiting is one of the warning symptoms	5 (1.2%)	38 (8.9%)	114 (26.8%)	167 (39.2%)	102 (23.9%)	3.76	0.956
Abdominal Pain is one of the warning symptoms	3 (0.7%)	58 (13.6%)	151 (35.4%)	123 (28.9%)	91 (21.4%)	3.57	0.995
Abdominal Fullness is one of the warning symptoms	14 (3.3%)	68 (16%)	154 (36.2%)	121 (28.4%)	69 (16.2%)	3.38	1.039

**Table 6:** Descriptive analysis of knowledge about risk factors and symptoms of gastric cancer

Variable	Mean ± SD	Min- Max
Knowledge about risk factors of gastric cancer	72.9± 11.4	22.5- 100
Knowledge about symptoms of gastric cancer	75.4 ± 14.4	36.7- 100

The differences in participants' knowledge of gastric cancer risk factors are illustrated in Table 7. A statistically significant difference was observed between males' (74.4) and females' (71.9) mean knowledge (t=2.203, p-value= 0.028). Regarding age, the results demonstrate statistically significant differences in the mean of the knowledge with respect to age, with the age group (less than or equal to 20 years) having a higher mean knowledge of 76.6 than the age groups (31-40 years) and (41-50 years) (F=7.27, p-value= 0.000). With regard to occupation, students have a higher knowledge (77.3) than all other groups which is statistically significant (F=11.98, p- value= 0.000). In addition, working or studying in a medical field affected their knowledge in that those working or studying in a medical field have a higher knowledge (77.3) than those who do not (70.8), and the difference is statistically significant (t=5.79, p- value= 0.000). Lastly, monthly income affects the knowledge about risk factors of gastric cancer significantly in that participants having an income of less than 5000 have a higher knowledge of 74.4 than the two groups (5000-10000) and (10000-20000), who have percentages of 70.6, and 71.3 respectively (F=3.29, p- value= 0.021). On the other hand, the result shows that education does not affect the participants’ knowledge about risk factors of gastric cancer as p-value>0.05

**Table 7:** Differences in participants’ knowledge about risk factors of gastric cancer considering demographic characteristics *Statistically significant

Variable	N	knowledge about risk factors Mean± SD	test	value	P-value
Gender					
male	184	74.4±12	t	2.203	0.028*
female	242	71.9±10.9			
Age					
<=20years	75	76.6±12.9	F	7.27	0.000*
21-30 years	170	74.6±11.6			
31-40years	65	69.9±11.5			
41-50years	69	68.2±8.7			
>50years	47	72.7±9.1			
Education					
Illiteracy	4	68.3±6.3	F	0.406	0.804
elementary school	8	71.4±10			
intermediate school	8	73.3±11.8			
high school	112	73±11.5			
College or above	294	74.1±3.4			
Occupation					
Student	178	77.3±10.9	F	11.98	0.000*
Employed	111	69.9±11.7			
Unemployed	91	69.4±10.5			
Retired	36	71.3±9.6			
Freelancer	10	69.9±9.9			
Working or studying in a medical field					
yes	144	77.3±11.1	t	5.79	0.000*
no	282	70.8±11			
Monthly income					
less than 5000	244	74.4±11.8	F	3.29	0.021*
5000-10000	80	70.6±10.9			
10000-20000	88	71.3±10.6			
more than 20000	14	71.9±11.7			
less than 5000	244	73±11.5			

The differences in participants' knowledge of gastric cancer risk factors based on their family and medical histories are illustrated in Table 8. The results revealed no statistically significant differences in risk factor knowledge for the three variables examined, as all p-values are greater than 0.05.

**Table 8:** Differences in participants’ knowledge about risk factors of gastric cancer considering medical and family histories.

Variable	N	knowledge about risk factors Mean± SD	test	value	P-value
Do you have any disease of the upper or lower GI tract “peptic ulcer, chronic gastritis, inflammatory bowel diseases etc.					
yes	66	72±11.1	t	-0.788	0.431
no	360	73.2±11.5			
Past Medical History of Gastric Cancer					
yes	3	71.7±8.3	t	-0.198	0.843
no	423	73±11.5			
Family History of Gastric Cancer					
yes	21	73.7±11.8	t	0.294	0.769
no	405	72.9±11.5			

Table 9 depicts a multivariate analysis to predict factors influencing participants' knowledge of gastric cancer risk factors. Five variables with a p-value less than or equal to 0.15 in the univariate analysis are included in the model. These are (gender, age, occupation, working or studying in a medical field, and monthly income). The model indicates that "studying or working in the medical field" is the only variable that affects their knowledge, as participants studying or working in a medical field increase their knowledge of risk factors by 43.89 % (B= 4.389, p-value= 0.002)

**Table 9:** Multivariate analysis, prediction of factors that affect knowledge about risk factors of gastric cancer.

	Unstandardized Coefficients		Standardized Coefficients	t	Sig.
	B	Std. Error	Beta		
(Constant)	78.478	2.637		29.761	0.000
Gender	-2.104	1.095	-0.091	-1.922	0.055
Age	-0.101	0.603	-0.011	-0.167	0.867
Occupation	-1.165	0.687	-0.111	-1.696	0.091
Do you work \ Study in a medical Field?	4.389	1.419	0.181	3.094	0.002*
Monthly income	-0.620	0.695	-0.049	-0.892	0.373

Differences in the participants’ knowledge of symptoms of gastric cancer are illustrated in Table 10. Regarding age, the results show statistically significant differences in the mean of knowledge with respect to age in that individuals in the age group of 41-50 years have a lower knowledge (69.4) than the participants in the three age groups <=20 years, 21-30 years, and >50years who have a knowledge score of 77.4, 77.4, and 75.7 respectively (F=4.51, p- value= 0.001). With regard to occupation, students have a higher knowledge (77.3) than all other groups which is statistically significant (F=11.98, p-value= 0.000). In addition, working or studying in a medical field affects their knowledge in that those working or studying in a medical field have a higher knowledge (77.3) than those who do not (70.8), and the difference is statistically significant (t=5.79, p-value= 0.000). Lastly, monthly income affects the knowledge about risk factors of gastric cancer significantly in that participants having income (less than 5000) have a higher knowledge of 74.4 than the two groups of 5000-10000 and 10000-20000, who have percentages of 70.6, and 71.3, respectively (F=3.29, p-value= 0.021). On the other hand, the results show that gender, education, and monthly income of more than 20000 do not affect the participants’ knowledge about symptoms of gastric cancer as all p-values > 0.5.

**Table 10:** Differences in participants’ knowledge about symptoms of gastric cancer considering demographic characteristics. *Statistically significant

Variable	N	knowledge about symptoms Mean± SD	test	value	P-value
Gender					
male	184	76.5±14.7	t	1.431	0.153
female	242	74.5±14.1			
Age					
<=20years	75	77.4±14.6	F	4.512	0.001*
21-30 years	170	77.4±15.1			
31-40years	65	73.8±14.2			
41-50years	69	69.4±13.3			
>50years	47	75.7±10.6			
Education					
Illiteracy	4	84.2±12.6	F	1.526	0.194
elementary school	8	64.6±6.7			
intermediate school	8	74.2±13.5			
high school	112	75.5±13.9			
College or above	294	75.5±14.7			
Occupation					
Student	178	77.3±13.8	F	11.98	0.000*
Employed	111	73.3±14.4			
Unemployed	91	70.8±14.4			
Retired	36	73.6±11.7			
Freelancer	10	68.3±13.8			
Working or studying in a medical field					
yes	144	77.3±12.7	t	5.79	0.000*
no	282	70.8±14.4			
Monthly income					
less than 5000	244	74.4±14.7	F	3.29	0.021*
5000-10000	80	70.6±13.5			
10000-20000	88	71.3±14.5			
more than 20000	14	77.1±13.1			

Differences in the participants’ knowledge of symptoms of gastric cancer with respect to their family and medical histories are illustrated in Table 11. The results revealed no statistically significant differences in the knowledge about symptoms for the three examined variables, as all p- values are > 0.05.

**Table 11:** Differences in participants’ knowledge about symptoms of gastric cancer considering medical and family histories.

Variable	N	knowledge about symptoms Mean± SD	test	value	P-value
Do you have any disease of the upper or lower GI tract “peptic ulcer, chronic gastritis, inflammatory bowel diseases etc.					
yes	66	75.9±12.3	t	0.306	0.760
no	360	75.3±14.8			
Past Medical History of Gastric Cancer					
yes	3	90±14.5	t	1.771	0.220
no	423	75.3±14.4			
Family History of Gastric cancer					
yes	21	76.3±14.3	t	0.322	0.747
no	405	75.3±14.4			

Table 12 illustrates the multivariate analysis used to predict the factors influencing participants' knowledge of the symptoms of gastric cancer. In the model, three predictors with a p-value of 0.15 in the univariate analysis are included. These are age, occupation, and working/studying in a medical field. Two variables affect the participants' knowledge of symptoms, according to the model. First: "studying or working in a medical field" in that participants studying or working in a medical field increase their knowledge of symptoms by 66.8% (B= 6.68, p-value = 0.000) Second: occupation, which differs in symptom knowledge by 16.9% (B = 1.69, p-value= 0.049).

**Table 12:** Multivariate analysis, prediction of factors that affect knowledge about symptoms of gastric cancer *Statistically significant

	Unstandardized Coefficients		Standardized Coefficients	t	Sig.
	B	Std. Error	Beta		
(Constant)	75.111	2.348		31.983	0.000
Age	0.549	0.684	0.048	0.803	0.422
Occupation	-1.698	0.862	-0.128	-1.971	0.049*
Do you work \ study in a medical field?	6.688	1.784	0.220	3.750	0.000*

**Figure 1:**
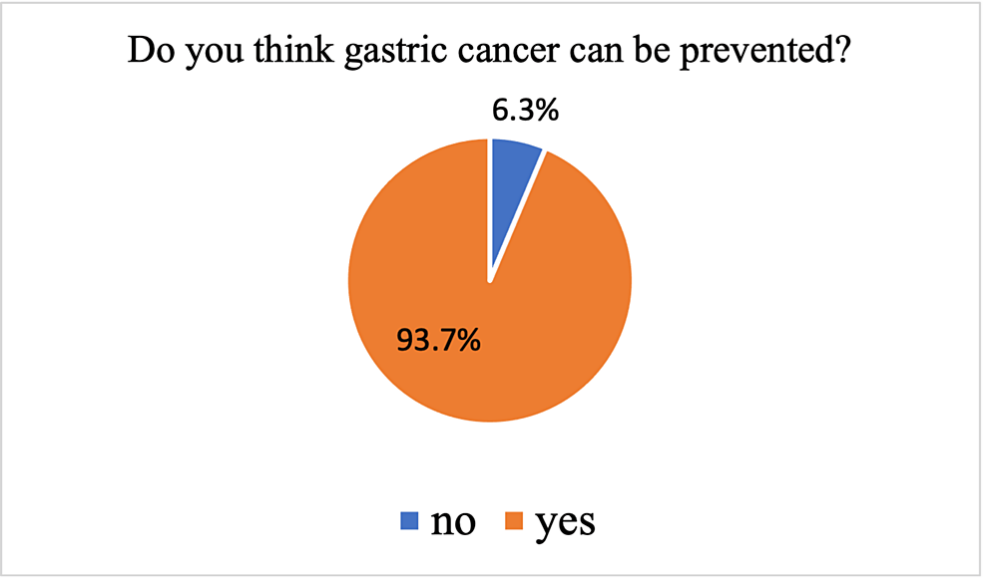
Participants’ perception of prevention of gastric cancer

**Figure 2:**
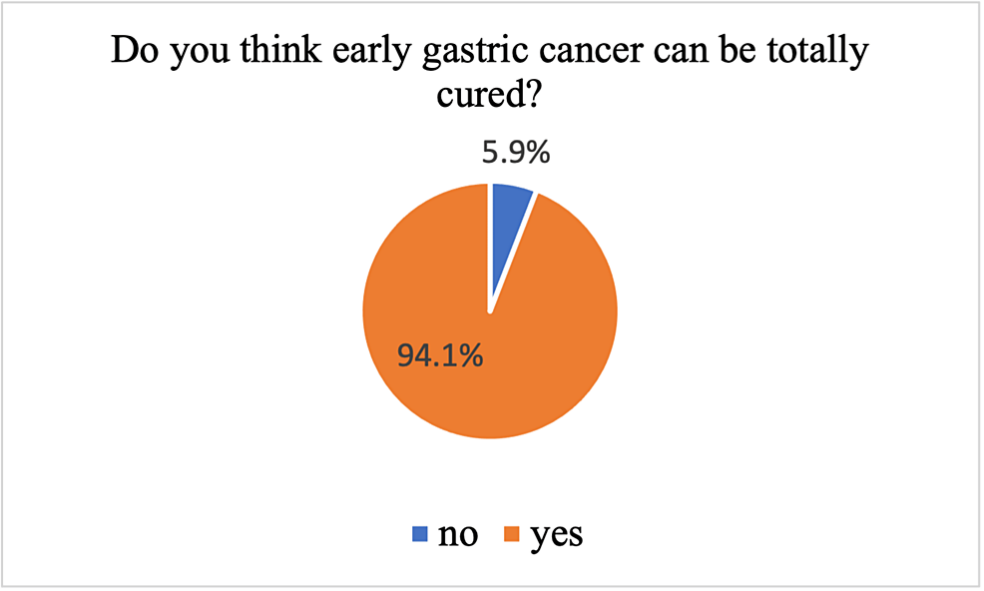
Participants’ perception of the curability of gastric cancer

Figure 1 and Figure 2 show the participants' perception of the prevention and cure of gastric cancer. Most of the participants think that gastric cancer could be prevented (93.7%) and cured (94.1%).

## Discussion

Gastric cancer has two primary subtypes, adenocarcinoma and mucosa-associated-lymphoid tissue (MALT) lymphoma [14]. It is the fifth most prevalent cancer in the world and is more prevalent in males [15]. Gastric cancer is the fourth most prevalent cancer among men and the fifth most prevalent cancer among women in Saudi Arabia [16,17]. This study was undertaken in Al-Baha City, Saudi Arabia, to measure the general population's knowledge and awareness regarding stomach cancer. The study involved 426 people recruited randomly from various city neighborhoods.

In the current study, few participants reported a history of upper or lower gastrointestinal (GI) tract disease, with peptic ulcer, chronic gastritis, and inflammatory bowel disease being the most prevalent. Little is known about the prevalence of GI tract diseases in Saudi Arabia [18]. However, several studies have attempted to assess the prevalence of specific types of GI tract diseases, such as the study by Albaqawi et al., which found that the prevalence of peptic ulcer and duodenal ulcers among the general population in the Arar region of Saudi Arabia was 16.2% and 5.6%, respectively [19]. However, these findings are concerning because these disorders have been linked to an increased risk of developing stomach cancer [20]. Also prevalent in the research population were gastroesophageal reflux disease (GERD) and chronic gastritis, with *H. pylori* and stomach ulcers accounting for a large number of cases. Infection with *H. pylori* is a well-known risk factor for gastric cancer [21], and its eradication has been demonstrated to lower the occurrence of this illness. Similarly, stomach ulcers can cause chronic inflammation and damage to the lining of the stomach, which increases the chance of developing gastric cancer [21]. Almost all of the individuals in the study had no prior medical history of stomach cancer, and only a tiny percentage had a family history of the disease. Overall, the study emphasizes the need of identifying and treating gastric cancer risk factors, such as *H. pylori* infection and persistent inflammation of the stomach lining.

According to the findings of the current study which was conducted in Al-Baha City, Saudi Arabia, participants had a firm understanding of the most generally recognized risk factors for stomach cancer. A family history of gastric cancer, a previous medical history of gastric cancer, and a stomach ulcer were all found as possible risk factors. Smoked food consumption was also found as a risk factor, though to a lower amount.

Heavy alcohol intake and smoking have been connected with an increased risk of developing gastric cancer [22,23]. Family history of gastric cancer and a past medical history of the disease are also known risk factors since they reflect a genetic predisposition or exposure to risk factors in the past [24,25]. Patients with stomach cancer showed a 2- to 3-fold higher incidence of a family history, indicating that a family history is an independent risk factor [26]. In addition to being a well-established risk factor for gastric cancer [27], stomach ulcer, especially when induced by *H. pylori* infection, is also a well-established risk factor for gastric cancer. The finding of smoked food consumption as a risk factor is intriguing, as it shows that the traditional preservation method of smoking food may contribute to the development of stomach cancer. The process of smoking can generate carcinogenic chemicals, such as polycyclic aromatic hydrocarbons, which can raise the risk of cancer [28]. However, similar to other prior research [29,30], many of the participants in the current study were unaware of the considerable link between *H. pylori* and stomach cancer. The outcomes of this study indicate that the general public of Al-Baha City has a good knowledge of the most generally recognized risk factors for stomach cancer. To study the prevalence and importance of this risk factor in Saudi Arabia and other populations, additional research is necessary. Educational programmes and campaigns can play a vital role in raising awareness of these risk factors and promoting the adoption of healthy lifestyle choices in order to lower the risk of getting stomach cancer.

According to the findings of the current study, the most commonly known signs of gastric cancer among the general population are gastrointestinal bleeding, abdominal mass, weight loss, recurrent nausea and vomiting, and abdominal discomfort. These findings are consistent with previous research on the signs of stomach cancer. Gastrointestinal bleeding is a common symptom of gastric cancer, which is caused by the erosion of blood vessels in the stomach by malignant cells [31,32]. This symptom might create stools that are dark or bloody, which can be difficult to identify without medical assistance [33]. Abdominal mass, on the other hand, may indicate an advanced stage of gastric cancer, in which a palpable mass can be felt in the abdomen due to the growth of the tumor [34,35]. Another typical sign of stomach cancer is weight loss, which is caused by a lack of appetite and trouble eating, which can result in malnutrition [36,37]. The restriction of the stomach's exit by the tumor, which causes an accumulation of food and fluid [38], can also cause recurrent nausea and vomiting. A common sign of gastric cancer is abdominal pain, which can be caused by the tumor's invasion of the stomach wall or nearby organs [39,40]. The general community must be aware of these signs, as they are critical for early detection and diagnosis of stomach cancer. It is essential to note, however, that similar symptoms can also be caused by other disorders, and a complete medical evaluation is required for an appropriate diagnosis.

Overall, the current study revealed that the participants had a moderate understanding of stomach cancer's risk factors and warning symptoms. This is comparable to the findings of previous research [41] indicating that the general community has a moderate understanding of the symptoms and risk factors of stomach cancer. While the general community in Al-Baha City has some understanding of the risk factors and symptoms of stomach cancer, there is opportunity for improvement according to our data. It is vital to highlight, however, that the participants' knowledge scores varied greatly, with some displaying a high level of understanding and others a lack of comprehension. The outcomes of this study underline the need for educational initiatives and campaigns to raise knowledge and comprehension of the risk factors and symptoms of stomach cancer. These initiatives can target both the general people and medical professionals in an effort to enhance disease identification and management. In addition, efforts should be made to increase access to screening and diagnostic services in the region so that stomach cancer can be detected earlier.

Our study shows that diverse subgroups of the population had significantly different levels of knowledge regarding the risk factors for stomach cancer. Males had a much higher mean knowledge score than females, and this difference was statistically significant. This finding is consistent with prior research indicating that men tend to have greater health-related knowledge and awareness than women [42]. However, several reports found that women were more knowledgeable about stomach cancer than men [13,43]. Regarding age, the study discovered that individuals aged 20 years and younger had a higher mean knowledge score than those aged 31 to 40 years and 41 to 50 years. This is consistent with the findings of earlier research, which found that middle-aged and older participants were less knowledgeable of gastric cancer than younger individuals [13], which is a cause for concern considering that stomach cancer is commonly diagnosed in individuals over the age of 40 [44]. This data shows that younger individuals are more likely to have access to health information and be responsive to instructional programmes. Students had access to educational resources and were exposed to health-related information, which contributed to their much better mean knowledge score than other occupational groups. Those who worked or studied in the medical area had a considerably higher mean knowledge score than those who did not, underscoring the significance of professional education and training in enhancing knowledge of health-related issues. Intriguingly, the study discovered that monthly income had a significant effect on knowledge regarding stomach cancer risk factors, with participants with lower income having a higher mean knowledge score than participants with greater income. However, this is not consistent with the findings of prior research, which indicated that patients with lower socioeconomic characteristics were more likely to have advanced cancer stages at the time of diagnosis [45,46], indicating a lack of awareness. This finding shows that individuals with a lower income may be more exposed to health education initiatives and more open to health-related information. The study indicated, however, that education had no significant effect on the participants' understanding of the risk factors of stomach cancer. This study implies that educational level alone may not be sufficient to increase health-related knowledge and awareness, and that other factors, such as access to health information and services, may play a more significant impact.

In addition, the results of the present study revealed that there were considerable disparities in awareness about the symptoms of stomach cancer across various subpopulations. The study indicated that individuals aged 41 to 50 years had a poorer average knowledge score on the signs of stomach cancer than participants of other ages. This data shows that persons in this age group may benefit from symptom-specific educational programmes. Regarding occupation, the study indicated that students and those working or studying in the medical area had considerably higher average knowledge scores about stomach cancer symptoms compared to other occupational categories. This research emphasizes the significance of exposure to health-related information for enhancing awareness of the signs of stomach cancer. In contrast, the study indicated that gender, education, and monthly income had no significant effect on participants' awareness of stomach cancer symptoms. This finding shows that initiatives designed to increase awareness of the signs of stomach cancer may need to be tailored to certain age and occupational groups.

Strengths and limitations

The main strength of our study is the high response rate, which could decrease the non-response bias. This high response rate may be attributed to experienced face-to-face interviewers and shorter survey length. Moreover, a face-to-face interview which has the highest response rates compared with other survey methods, was used in this study. Our study has several limitations. First, the participant's past medical and family history were self-reported, and hence, the possibility of recall bias could not be eliminated. Second, the majority of responders were highly educated. Therefore, our results may not apply to all different structures of the population. Moreover, the small sample size of our study may increase the chance of errors. Therefore, we recommend conducting similar studies with a larger sample size to confirm and generalize our results. 

## Conclusions

In conclusion, the study done in Al-Baha City, Saudi Arabia, provides useful insights into the general population's degree of knowledge and awareness regarding stomach cancer. The participants in the study demonstrated a modest degree of awareness of the risk factors and symptoms of stomach cancer, with significant variation between segments of the population. It also emphasized the significance of recognizing and controlling gastric cancer risk factors, such as *H. pylori* infection and chronic inflammation of the stomach lining. Educational programmes and campaigns can play a crucial role in raising awareness and comprehension of these risk factors and promoting the adoption of healthy lifestyle choices to lower the chance of getting gastric cancer. In addition, the study identified numerous subsets of the population who may benefit from focused educational initiatives, including those aged 41 to 50 and those in non-medical employment. The study emphasizes the significance of implementing targeted educational programmes to increase knowledge and awareness of stomach cancer among various subpopulations. In addition, the findings of the study highlight the need for enhanced awareness and screening programmes to diagnose stomach cancer at an early stage, especially in groups with a high incidence of risk factors. To develop effective prevention and care methods for this disease, additional study is required to examine the prevalence and risk factors of stomach cancer in Saudi Arabia and related populations.

## Appendices

**Table 13:** Appendix 1

Sociodemographic Data	
Age	Below 18 Years
	18-29 Years
	30-39 Years
	40-49 Years
	50-59 Years
Gender	Male
	Female
Nationality	Saudi
	Non-Saudi
Residence	Al-Baha City
	other
Marital status	Single
	Married
	Divorced
	Widowed
Educational level	Illiterate
	Elementary school
	Middle school
	High school
	College or above
Occupation	Employed
	Unemployed
	Freelancer
	Student
Do you work \ study in a medical field?	Yes
	No
Monthly income	Less than 5000 Ryals
	5000-10000 Ryals
	10000-20000 Ryals
	More than 20000 Ryals
Do you have any disease of the upper or lower GI tract ( Peptic ulcer, chronic gastritis….. etc. )	Yes
	No
If the answer to the previous question is yes, what’s the medical diagnosis?	To be written
Do you have a past medical history of Gastric cancer?	Yes
	No
Do you have a family history of gastric cancer?	Yes
	No

**Table 14:** Appendix 2

From the listed variables below, choose the gastric cancer risk factors that you agree\ disagree upon.					
variable	Strongly agree	Agree	Neutral	Disagree	Strongly disagree
Male sex					
Older age					
H. pylori infection					
Stomach ulcer					
Previous stomach surgery					
Atrophic gastritis					
Family history of gastric cancer					
Salty diet					
Consumption of pickled food					
Consumption of smoked food					
Irregular\unhealthy diet					
Frequent eating of leftovers					
Frequent midnight snacking					
Smoking “ cigarette\ Shisha					
Alcohol consumption					
Stress					

**Table 15:** Appendix 3

From the listed variables below, choose the gastric cancer warning symptoms that you agree\ disagree upon.					
variable	Strongly agree	agree	Neutral	disagree	Strongly disagree
GI Bleeding					
Recurrent Nausea and vomiting					
Weight loss					
Abdominal lump					
Abdominal pain					
Abdominal fullness					

**Table 16:** Appendix 4

Perception on the curability and preventability of gastric cancer	
Do you think gastric cancer can be prevented?	Yes
	No
Do you think early gastric cancer can be totally cured?	Yes
	No
